# High-recovery visual identification and single-cell retrieval of circulating tumor cells for genomic analysis using a dual-technology platform integrated with automated immunofluorescence staining

**DOI:** 10.1186/s12885-015-1383-x

**Published:** 2015-05-06

**Authors:** Daniel E Campton, Arturo B Ramirez, Joshua J Nordberg, Nick Drovetto, Alisa C Clein, Paulina Varshavskaya, Barry H Friemel, Steve Quarre, Amy Breman, Michael Dorschner, Sibel Blau, C Anthony Blau, Daniel E Sabath, Jackie L Stilwell, Eric P Kaldjian

**Affiliations:** 1 RareCyte, Inc, Seattle, WA USA; 2 Medical Genetics Laboratories, Baylor College of Medicine, Houston, USA; 3 Department of Pathology, University of Washington, Washington, USA; 4 Rainier Hematology-Oncology, Northwest Medical Specialties, Washington, USA; 5 Center for Cancer Innovation, University of Washington, Washington, USA; 6 Departments of Laboratory Medicine and Medicine, University of Washington, Washington, USA

## Abstract

**Background:**

Circulating tumor cells (CTCs) are malignant cells that have migrated from solid cancers into the blood, where they are typically present in rare numbers. There is great interest in using CTCs to monitor response to therapies, to identify clinically actionable biomarkers, and to provide a non-invasive window on the molecular state of a tumor. Here we characterize the performance of the AccuCyte® – CyteFinder® system, a comprehensive, reproducible and highly sensitive platform for collecting, identifying and retrieving individual CTCs from microscopic slides for molecular analysis after automated immunofluorescence staining for epithelial markers.

**Methods:**

All experiments employed a density-based cell separation apparatus (AccuCyte) to separate nucleated cells from the blood and transfer them to microscopic slides. After staining, the slides were imaged using a digital scanning microscope (CyteFinder). Precisely counted model CTCs (mCTCs) from four cancer cell lines were spiked into whole blood to determine recovery rates. Individual mCTCs were removed from slides using a single-cell retrieval device (CytePicker™) for whole genome amplification and subsequent analysis by PCR and Sanger sequencing, whole exome sequencing, or array-based comparative genomic hybridization. Clinical CTCs were evaluated in blood samples from patients with different cancers in comparison with the CellSearch® system.

**Results:**

AccuCyte – CyteFinder presented high-resolution images that allowed identification of mCTCs by morphologic and phenotypic features. Spike-in mCTC recoveries were between 90 and 91%. More than 80% of single-digit spike-in mCTCs were identified and even a single cell in 7.5 mL could be found. Analysis of single SKBR3 mCTCs identified presence of a known TP53 mutation by both PCR and whole exome sequencing, and confirmed the reported karyotype of this cell line. Patient sample CTC counts matched or exceeded CellSearch CTC counts in a small feasibility cohort.

**Conclusion:**

The AccuCyte – CyteFinder system is a comprehensive and sensitive platform for identification and characterization of CTCs that has been applied to the assessment of CTCs in cancer patient samples as well as the isolation of single cells for genomic analysis. It thus enables accurate non-invasive monitoring of CTCs and evolving cancer biology for personalized, molecularly-guided cancer treatment.

**Electronic supplementary material:**

The online version of this article (doi:10.1186/s12885-015-1383-x) contains supplementary material, which is available to authorized users.

## Background

Cancer metastasis accounts for 90% of cancer deaths [1]. Circulating tumor cells (CTC) are malignant cells that migrate from a cancer into the bloodstream; most CTCs die, but some exit the circulation to develop into metastases [2]. High numbers of CTC are associated with shorter overall and progression free survival [3-5]. CTCs, however, are rare – it is typical for one CTC to be present for every million white blood cells or more – and thus detecting and measuring CTC requires highly sensitive technology.

Platforms for CTC identification have been developed based on size, protein expression, or other physical characteristics (reviewed in [6]). Currently, the only FDA-cleared platform for CTC enumeration is the CellSearch® system (Veridex, Raritan, NJ, USA), and is used for monitoring CTC in patients with colorectal, breast, and prostate cancer. This system is based on automated immuno-magnetic capture of EpCAM expressing cells, followed by staining for DNA and cytokeratin to verify that captured cells are nucleated and epithelial in origin. An exclusionary stain for CD45 is included to prevent false positive identification of white blood cells that may be non-specifically captured. False negatives are an acknowledged weakness of immuno-magnetic capture, which will not identify CTCs that express low levels of the capture antigen. Other technologies for CTC analysis currently under development include other immunomagnetic positive or negative selection methods, microfluidic chips, filters, isolation based on cell deformability or cell density, and dielectrophoretic separation. Although there are advantages to each technology, there are also limitations. Microfluidic chips and filters that fractionate by size will not capture small CTCs. Most technologies do not provide high-resolution visualization of cells. Often sensitive technologies are not specific, and vice versa. Some require red blood cell lysis, which may damage cells. Finally, the ability to robustly retrieve individually identified cells within a practical workflow remains elusive.

The use of information from CTCs for therapeutic decision-making is in its infancy. There is great interest in exploiting CTCs as a window on the molecular state of a tumor, since understanding the evolutionary path of a cancer may predict resistance before overt clinical progression, potentially allowing for the pre-emptive selection of a more effective therapy. An ideal CTC analysis platform would provide unambiguous morphology for definitive CTC identification, comprehensive CTC enumeration for monitoring a patient’s response to therapy, flexible characterization of biomarkers (including drug targets), and also enable isolation of CTCs for molecular analyses.

We characterize here the performance of the AccuCyte® – CyteFinder® system: a comprehensive, reproducible and highly sensitive dual-technology platform for collecting, identifying and analyzing CTCs, that employs two complementary technologies that surround a staining step using an automated immunohistochemistry instrument. The AccuCyte system – “front end” – is based fundamentally on the density of CTCs, which is within the range of the buffy coat. However, it is differentiated from existing density-based methods that separate the buffy coat from red blood cells and plasma by use of a unique separation tube and collector device, which allows virtually complete harvesting of the buffy coat into a small volume for application to a microscopic slide without cell lysis or wash steps, a potential source of CTC loss. The CyteFinder system – “back end” – is an automated scanning digital microscope and image analysis system that presents high-resolution images of candidate cells stained with well-characterized markers before definitive classification as a CTC. CyteFinder includes an integrated device (CytePicker™) for CTC retrieval that is mechanically precise and compatible with recently developed advanced genomic analysis methods for single CTCs.

## Methods

### Blood sample collection for spike-in experiments

Blood samples were collected from healthy volunteers at Rainier Clinical Research Center according to a protocol approved by Quorum Review institutional review board (IRB, Seattle, WA, USA). Approximately 40 mL was collected from healthy volunteers into anticoagulant EDTA Vacutainer® tubes (Becton-Dickinson) with a proprietary preservative (RareCyte, Seattle, WA, USA) and 20 mL was collected from cancer patients into CellSave® tubes (Veridex, Raritan, NJ, USA).

### Tissue culture cells and model CTC (mCTC) spike-in experiments

LNCaP and PC3 (Prostate), A549 (lung), and MCF7 and SKBR3 (breast) cancer cell lines used as model CTCs (mCTC) were all obtained from American Type Culture Collection (ATCC, Manassas, VA, USA). LNCaP, PC3, SKBR3, and A549 cell lines were maintained in RPMI 1650 medium and MCF7 cell lines were maintained in DMEM medium. Media were supplemented with 10% FBS.

For percent recovery determination, nuclei or mitochondria of live mCTCs were fluorescently labeled with Hoechst 33342 or Mitotracker Red (Life Technologies), respectively, and drawn into a glass capillary tube (VitroTube, Mountain Lakes, NJ, USA). The cells within the VitroTube were then scanned and counted using a DeltaVision fluorescent microscope (GE, Issaquah, WA, Additional file 1: Figure S1). Cells were expelled into 7.5 mL of blood by flushing the VitroTube with PBS and then rescanning the tube for cells that were not expelled to obtain the net precise count of the cells added to the blood. On the order of 100 cells (range ~70 – 200) from each mCTC cell line were spiked into 5 different blood samples and then the sample was processed as described in the next section.

For low mCTC detection experiments, freshly prepared Hoechst 33342 labeled PC3 cells were suspended at approximately 10,000 cells per mL and then pipetted into a well of a multi-chambered glass slide that allowed cells to remain in solution. The chambered slide was then imaged on the CyteFinder® fluorescent microscope (RareCyte, described below). Individual PC3 cells were drawn into a ceramic-tipped needle using the integrated CytePicker™ (RareCyte, described below) and deposited into a PCR tube. The contents of the PCR tube were then transferred into a blood sample by washing with PBS. Alternatively, the contents of the CytePicker needle were deposited into a separate sorting well on the chambered slide. The sorting well was then imaged to determine an accurate count of the number of PC3 cells deposited and the contents of the well were washed into a blood sample with PBS. From 1 cell to 6 cells were spiked into 7.5 ml blood samples.

### Density enrichment and adherence of buffy coat to slides

**Figure 1:**
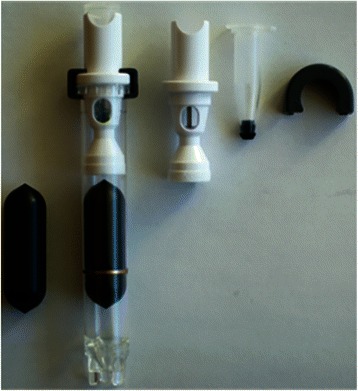
Components of the AccuCyte® system. From left to right is pictured the float; the entire assembly for separation and isolation of the buffy coat, including the Separation Tube with sealing ring; the EpiCollector®; the Transfer Tube with septum base that that is pierced by the EpiCollector needle to allow the flow of material from the Separation Tube to the Transfer Tube; and the clamp that secures the entire apparatus.

**Figure 2:**
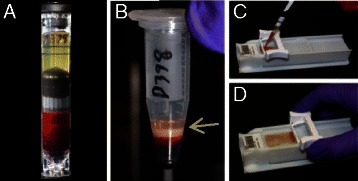
Isolation of buffy coat and spreading onto microscopic slides. **(A)** AccuCyte® Separation Tube and float after initial centrifugation to separate 7.5 mL blood sample into its component layers – plasma/buffy coat/red blood cells. **(B)** Isolated buffy coat in Transfer Tube after transfer centrifugation. Arrow indicates the buffy coat. **(C)** Addition of buffy coat mixture to slide. **(D)** Spreading cells onto glass slide using CyteSpreader™ device.

Each spiked blood sample (7.5mls) was added to an AccuCyte® Separation Tube (RareCyte) containing a lozenge-shaped float (Figure 1). The float is a hollow plastic cylinder with longitudinal ribs raised 75 microns on the surface to prevent contact of the float body with the inside wall of the tube, thereby providing channels for fluid movement during centrifugation. The leading and trailing ends of the float are rounded to reduce turbulence and shear forces during centrifugation and so prevent cell damage. The density of the float is adjusted to allow it to rest at the red blood cell – plasma interface (containing the buffy coat) after centrifugation, typically between 1.051 and 1.057 gm/mL (or specific gravity units, SG). Clinical samples were processed in the same way, without the addition of spiked in cells. The sample was centrifuged in a Beckman Allegra X-15R table top centrifuge with SX4750 swinging bucket rotor (Beckman Coulter, Indianapolis, IN) at 5250 relative centrifugal force (RCF) for 30 minutes. Centrifuge adaptors specially made to contain these tubes and floats (RareCyte) were used to allow a controlled expansion of the inner diameter of the tube while preventing over-expansion or rupture. Centrifugation separates the blood within the Separation Tube into a bottom layer of packed red blood cells (the hematocrit), a top layer of plasma, and the buffy coat layer of white blood cells and platelets that collects within space between the float and the wall of the tube where it is easily visualized since its surface area expands within the narrow space (see Figure 2).

After centrifugation the Separation Tube was removed from the centrifuge adaptors and placed into a CyteSealer® (RareCyte), which applies a brass ring clamp (CyteSeal) around the circumference of the tube at a position on the float below the buffy coat layer, to create a barrier seal between the tube and the float. After the seal was applied, the plasma was aspirated from the top of the float and approximately 4 ml of 1.793 gm/mL high-density retrieval (HDR) fluid was added to the tube. A collection device (EpiCollector®, RareCyte) was placed into the top of the Separation Tube. The EpiCollector has an inverted funnel that tapers to a 16 gauge needle oriented upwards. Excess HDR fluid was expelled from the needle as the EpiCollector was inserted, eliminating dead space within the EpiCollector. A Transfer Tube pre-filled with approximately 250 uL of HDR fluid was placed into the EpiCollector; the Transfer Tube has a rubber septum at its base that is pierced by the needle within the EpiCollector. The Separation Tube with inserted EpiCollector and Transfer Tube was centrifuged for 5 minutes at 500 RCF (Beckman Allegra® X-15R) resulting in the buoyant displacement of the buffy coat from the float into the Transfer Tube. The workflow is summarized in Additional file 2: Figure S2.

Adherence Solution (1000 ul, RareCyte) was added to the buffy coat in the collection tube and mixed. The sample was spread onto 8 SuperFrost® Plus slides (VWR) by pipetting 150 uL of the mixture onto a slide resting in a manual spreading device (CyteSpreader®, RareCyte) that was designed to evenly distribute the sample in a monolayer across a defined region of the slide without making contact with the slide and thus minimizing sample loss (see Figure 2).

### Immunofluorescence staining

Slides were dried for 30 minutes, fixed in 10% Neutral buffered formalin (NBF, Sigma Aldrich) for 1 hour, washed in PBS for 1 minute, and then incubated with 1 M Tris–HCl 10 minutes to neutralize the NBF. Slides were washed twice more with PBS and then stained using the Discovery Ultra automated slide staining system (Ventana Medical Systems, Tucson, Arizona, USA). Antigen retrieval was performed by heating the slides for 8 minutes at 90°C using buffer CC1. Slides were incubated with antibody to EpCAM (SPM491, Spring Bioscience, Pleasanton, CA, USA) diluted 1:100 for 32 minutes in a solution containing 2% goat serum and 2% BSA. Slides with A549 cells spiked into blood were incubated with EGFR antibody (Invitrogen, clone 31G7) at 1:100 in place of EpCAM. Goat anti-mouse secondary antibody conjugated to Alexa Fluor®647 (Life Technologies) was added at a 1:1000 dilution for 24 minutes in a 2% goat serum and 2% BSA solution. The slides were then incubated with Alexa Fluor® 488 labeled cytokeratin antibody (clones AE1 and AE3, 1:200 dilution, eBioscience, San Diego, CA, USA), Alexa Fluor® 488 labeled cytokeratin antibody (C11, 1:100 dilution, BioLegend, San Diego, CA, USA), and R-phycoerythrin (PE) labeled CD45 antibody (HI30, 1:100 dilution, BioLegend) for 48 minutes in a 2% mouse serum and 2% BSA solution. All antibodies and serum diluents were stored in Inline User-Fillable Dispensers (Ventana) at 4x working concentration and diluted into Reaction Buffer (Ventana). DAPI or Hoechst 33342 was also included in this last incubation at 5 ug/mL/mL. Washes were performed by the Discovery Ultra as per manufacturer’s protocol. After completion of staining slides were removed and placed in Reaction Buffer for 5 minutes and washed 5 times with distilled water, and once with PBS. Coverslips were applied using Fluoromount (Sigma Aldrich). Slides were dried for at least 1 hour at room temperature before scanning. For clinical samples, some slides were stained with Ki67 antibody (clone 7B11, 1:100 dilution, Invitrogen, Carlsbad, CA, USA ) using a similar protocol to that used for EpCAM staining, substituting Ki-67 for EpCAM.

### Clinical samples

Blood was collected from advanced breast, prostate and colorectal patients being followed at the Seattle Cancer Care Alliance according to a protocol approved by the Fred Hutchinson Cancer Research Center IRB. Blood was collected from a patient with triple-negative breast cancer as part of the ITOMIC study by the Center for Cancer Innovation at the University of Washington (clinicaltrials.gov identifier NCT01957514); the study protocol was approved by the Fred Hutchinson Cancer Research Center IRB. Appropriate informed consent was received from all cancer patients. Blood samples were processed onto slides and stained on the Discovery Ultra as described above.

### Automated image capture and analysis

After staining, slides were placed onto the CyteFinder digital scanning microscope to acquire fluorescent images. The microscope is oriented with the objective positioned below the sample. For each slide, the CyteFinder acquired 4-channel fluorescent images of 2542 discrete fields of view to cover the area on the slide where the sample was spread (Additional file 3: Figure S3). Individual fields of view overlap by approximately 50 μm on all sides to prevent obtaining partial images of cells on the borders of adjacent fields. A solid-state, LED illuminator (Lumencor, Beaverton, OR) was used to excite the fluorophores. Images were captured using a Coolsnap® EZ CCD camera (Photometrics, Tucson, AZ). Filters for excitation and emission were from the Brightline® product collection (Semrock, Rochester, NY). Low magnification scan images were acquired with a Nikon 10X 0.3NA objective (Nikon Instruments, Melville, NY) with a lateral resolution of 1.06 um. The high resolution images of revisited points were acquired with a Nikon 40X 0.6NA objective with a lateral resolution of 529 nm. Revisited points were imaged with a “stack” of images through the Z plane with 1um steps. The images were presented to the reviewer as individual z planes rather than projection images.

Images were analyzed for the presence of signal above background for each channel (except nuclear dye channel) using Analyzer image analysis software (RareCyte) that employs an adaptive auto-threshold algorithm. The primary detection was performed on the fluorescent channel corresponding to the cytokeratin (CK) label. The objects identified by their CK signal were then analyzed to determine their correlation with the CD45 label (a negative marker). Highly correlative objects were rejected as this indicated the presence of CD45 label on CK positive objects. Objects that are determined by the algorithm to be CK positive and CD45 negative were presented to the reviewer for classification (see next section). Objects to be classified are termed “glyphs” and are highlighted by a 200 × 200 pixel box.

### Review and cell classification

CyteMapper® is a review software system that presents glyphs to the reviewer as a row of 4 boxes showing each individual fluorescence channel as grayscale images with scalable brightness and contrast (Additional file 4: Figure S4). A later version of the viewer included a fifth box showing a color composite image of channels superimposed on one another. The reviewer can view the entire panel in which the glyph was found to determine its relationship to other cells in the sample and can zoom in on images to facilitate classification.

Objects were classified into three categories: (1) “Cell”, (2) “Not a Cell”, or (3) “Indeterminate” based on established criteria for cells of epithelial origin [7-9]. A “Cell” met all criteria for a CTC, including positive nuclear stain, a positive cytokeratin signal, and a negative CD45 signal. EpCAM or EGFR (for A549 mCTCs) were used as additional interpretive markers for classification of “Cell”. An “Indeterminate” object met a combination of criteria that may include positive signal in two of three channels and/or positive signal in the “negative” channel. “Not a Cell” is used for all other objects. A tally of the number of objects in each category was kept by the software and reported upon saving the reviewed file. Only objects classified as “Cell” were included in tallies of CTCs. The performance of CyteMapper review for the mCTC spike-in experiments was shared among three scientists with extensive experience in the investigation of CTCs and in the use of CyteMapper for the identification of epithelial cells.

### CTC enumeration comparison

Blood from 10 patients with advanced breast, prostate or colorectal cancer was evaluated in a clinical feasibility study. Two 7.5 mL specimens of blood were drawn from cancer patients at the same time; one was given to the University of Washington (UW) Medical Center clinical laboratory for CTC evaluation by CellSearch and the other to RareCyte for CTC evaluation by AccuCyte – CyteFinder. CTCs were counted by CellSearch according to manufacturer’s instructions (Janssen Diagnostics, Raritan, NJ) and by AccuCyte – CyteFinder as described above. CTCs identified by AccuCyte – CyteFinder met CellSearch criteria: positive staining for cytokeratin and nucleus and negative staining for CD45. Investigators at RareCyte were blinded to the CellSearch counts until after the results from both assays were documented and delivered to investigators at UW.

### Retrieval of individual mCTC from slides

Isolation of single cells from slides was performed with CytePicker that is integrated with CyteFinder (Additional file 5: Figure S5). CytePicker is a hydraulically controlled semi-automated single cell retrieval device that contains three critical parts: (1) needle with 22 um-bore ceramic tip, (2) pump capable of 200 pL droplet resolution, (3) precision Z-positioning system using a piezo-electric actuator. Imaging of the cells was performed with a 10x, 0.30NA objective through the slide (rather than through a coverslip) so that uncovered cells are accessible to the ceramic tipped needle above the slide. Chromatic aberrations are measured and compensated for in software prior to imaging so that all fluorescent channel images are appropriately co-registered.

SKBR3 mCTCs were spiked into blood, which was processed and stained as above for cytokeratin, EpCAM, CD45 and nuclear DNA. Samples that were used for individual cell retrieval were prepared without a coverslip. After CyteFinder scanning, the Imager3 software module used the data generated from the scan/analysis/review routine to create a list of coordinates of cellular locations on the slide. Individual cell locations were visited (and viewed at 40× objective magnification if desired) to verify that the candidate cell met CTC criteria described above. A droplet of PBS was deposited on the slide in the area of the cell of interest. Using the CytePicker software module, the needle was lowered to make contact with the sample surface. Using the piezo-actuated Z control, the operator directed the needle tip 20–30 μm past the surface of the sample to “cut” into the sample layer. A controlled circular movement (termed “wiggle”) with a diameter between 25 and 40 μm was directed by the Imager3 software to dislodge the cell from the surface of the slide into the needle tip. Removal of the cell was confirmed visually (see Additional file 6: Figure S6). The needle was then raised and the operator placed a PCR tube under the needle. A volume of 2 μL was then dispensed into the bottom of the PCR tube and the sample was immediately frozen at -80C.

### Laboratory workflow

The workflow for the process of CTC collection, slide preparation and staining, scanning and image analysis and individual cell retrieval involves automated and manual steps. The times required for each step, and the proportion of “hands-on” time for the process that was current at the time of the submission of the revised manuscript is listed in Table 1. The total laboratory time for processing a single sample is less than 7 hours, with hands-on time of about 1 hour. Additional samples may be batch processed in the AccuCyte and automated staining steps with minimal additional hands-on time.

**Table 1:** **AccuCyte – CyteFinder laboratory workflow (in minutes)**

Step	Time/hands-on time
AccuCyte Collection	70/10
Automated Staining	210/15
CyteFinder Scanning	105/15
Image Review/CTC Confirmation	15/15
Total AccuCyte - CyteFinder	**400/55**
CytePicker cell retrieval (per cell)	2 - 3

### Whole genome amplification and molecular analysis of mCTC

After thawing individually picked SKBR3 cells at room temperature, the cells were lysed and genomes amplified with the Ampli1 WGA procedure according to manufacturer’s instructions (Silicon BioSystems, Bologna, Italy). Approximately 1 μL of the WGA reaction product was used for amplification of the TP53 gene that encodes the region of the protein containing the p.R175H mutation. Nested PCR primers were designed from the NCBI human reference genomic sequence and amplified from ch17:7577987–7578592 for the outer primers (5′-CCCTGACTTTCAACTCTGTCTC-3′ and 5′-AGGCCCTTAGCCTCTGTAA-3′) and ch17:7578281–7578503 for the inner primers (5′-GTGCAGCTGTGGGTTGATT-3′ and 5′-GGGCCAGACCTAAGAGCAAT-3′) using Primer3 software [10,11]. The amplicon generated from the outer primer set was 606 bp and from the inner primer set was 224 bp. Approximately 1 μL of sample from the WGA product was transferred into a PCR tube with 2X PCR reaction mix (New England Biolabs, Ipswich, MA, USA), 0.5 μM of each primer, and water was mixed and placed into a thermal cycler (Thermo Fisher Scientific). Thermal cycling conditions were as follows: (1) incubation at 94°C for 7 minutes, (2) 30 cycles of 94°C for 30 seconds, 60°C for 30 seconds and 72°C for 30 seconds, (3) final extension at 72°C for 7 minutes. Samples were held at 4°C until they were analyzed by gel electrophoresis. After PCR, the presence of the 224 bp amplicon was confirmed by loading a portion of the reaction onto a 2% agarose gel, and staining with SYBR® safe (Invitrogen) and comparing its migration to a DNA size standard.

The resulting amplicon was purified from primers using the DNA Clean & Concentrator (Zymo Research, Irvine, CA, USA) according to manufacturer’s instructions. Approximately 1 ng of amplicon was mixed with sequencing primer (inner PCR primers) and BigDye® Terminator sequencing reactions (Life Technologies) were performed according to manufacturer’s directions. Reactions were run on a 3730XL DNA Analyzer (ThermoFisher Scientific). Sequences were analyzed for the presence of the nucleotide mutation that defines p.R175H (c.524G > A).

### Array CGH

WGA products from single SKBR3 cells were analyzed by array CGH using oligonucleotide-based SurePrint G3 Human CGH 4x180K arrays from Agilent Technologies (Santa Clara, CA) as described previously [12]. Briefly, one microgram of WGA DNA was labeled per hybridization. Since the WGA products ranged in size from 100 bp to 1 kb, it was not necessary to perform DNA fragmentation before labeling. Test DNAs were labeled with dCTP-Cy5 and reference DNAs were labeled with dCTP-Cy3, for 2 hours at 37°C using a Spectral Labeling Kit (Perkin Elmer, Boston, MA). Unincorporated nucleotides were removed using a MultiScreen-PCRμ96 Filter Plate (Millipore, Billerica, MA). Hybridizations were carried out at 65°C for 40–72 hours to enhance the binding of WGA DNA, after which they were washed and scanned using an Agilent Microarray Scanner (PN G2565BA). Data was extracted using Agilent’s Feature Extraction software (version 9.5.3.1) and was analyzed using Agilent CytoGenomics Edition 2.5.8.11. The DNA used as a reference for each single lymphoblast cell WGA product was a pool of WGA DNA from multiple (5–10 single cell) WGA reactions from either male or female lymphoblast reference cell lines. Gender-mismatched references were used unless otherwise indicated.

Slides were scanned into image files using the Agilent G2565 Microarray Scanner. Scanned images were quantified using Agilent Feature Extraction software (v10.10.0.23). Text file outputs containing quantitative data were imported into the Agilent CytoGenomics software (version 2.5.8.11). Data were analyzed using the Aberration Detection Method 2 (ADM2) statistical algorithm at a threshold of 6.0 to identify genomic intervals with copy number changes. To reduce false positive calls, a filter was applied to define the minimum log2 ratio (0.25), the minimum size (100 kb) and the minimum number of probes (100) in a CNV interval. The Derivative Log Ratio Spread (DRLS), a measure of probe to probe noise calculated by the CytoGenomics software, was used as a performance measure for hybridization quality.

The karyotype of SKBR3 for reference comparison is found at this this website: http://old-www.path.cam.ac.uk/~pawefish/BreastCellLineDescriptions/sk-br-3.htm.

### Whole exome sequencing

A DNA fragment library was constructed from WGA products from individual SKBR3 cells picked from whole blood spike-in samples using a modified version of the NEBNext (New England Biolabs) protocol. Libraries were enriched using the SeqCap EZ Exome v3 capture system (Roche NimbleGen) for the coding portion of the genome. The target includes all coding content from the CCDS, RefSeq and miRBase databases. Paired-end (100 base pair) sequencing of enriched libraries was performed using a HiSeq 2500 system with TruSeq v3 chemistry (Illumina) with a read depth of 15 – 30x. The resulting reads were aligned to the genome human reference (hg19) using BWA (Burrows-Wheeler Aligner) [13] and variants called with GATK (Genome Analysis Toolkit) [14,15].

## Results

### Recovery of spiked-in mCTC from whole blood

**Figure 3:**
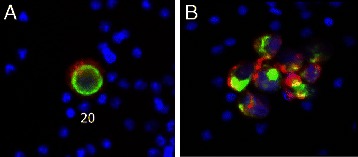
Fluorescently stained model circulating tumor cells collected and imaged using the AccuCyte® – CyteFinder® system. **(A)** A549 mCTC stained with antibody to EGFR (red), cytokeratin (green), and nuclear dye (blue). **(B)** Cluster of LnCAP mCTCs stained with antibody to EpCAM (red), cytokeratin (green), and nuclear dye (blue). Cells imaged at scanning 10X objective magnification.

Four cancer cell lines representing breast, prostate and lung cancer were used for mCTC recovery experiments. Approximately one hundred tumor cells (range 70 – 210) were precisely counted in capillary tubes and then spiked into 7.5 mL of whole blood. After cells were spiked into blood, the sample was centrifuged in the AccuCyte Separation Tube resulting in separation of the blood into its component layers – plasma, buffy coat and red blood cells (Figure 2A). The buffy coat was collected as described in Methods by centrifugation into the Eppendorf Transfer Tube (Figure 2B). Cells collected in the Transfer Tube were spread onto a glass slide with the CyteSpreader (Figure 2 C and D), and stained on the Discovery Ultra automated staining system, using antibodies to the epithelial antigens cytokeratin and EpCAM (EGFR in the case of A549), the leukocyte antigen CD45, and a DNA dye (Hoechst 33342 or DAPI). Epithelial staining of the mCTC distinguished them from cells normally within the blood (Figure 3). Slides were imaged on CyteFinder. After scanning, the images were analyzed by CyteMapper software, designed to identify cells by user-defined criteria including signal intensity, object size and cellular morphology. A blinded reviewer, different from the person who performed the spike-in, reviewed candidate cells by examining for positive epithelial antigen staining, presence of a nucleus, morphology consistent with a tumor cell, and absence of staining for CD45. Cells that met these criteria were counted as mCTC. Objects could be viewed in greater detail within the software if desired. Representative images of A549 and LNCaP mCTC are shown in Figure 3.

**Figure 4:**
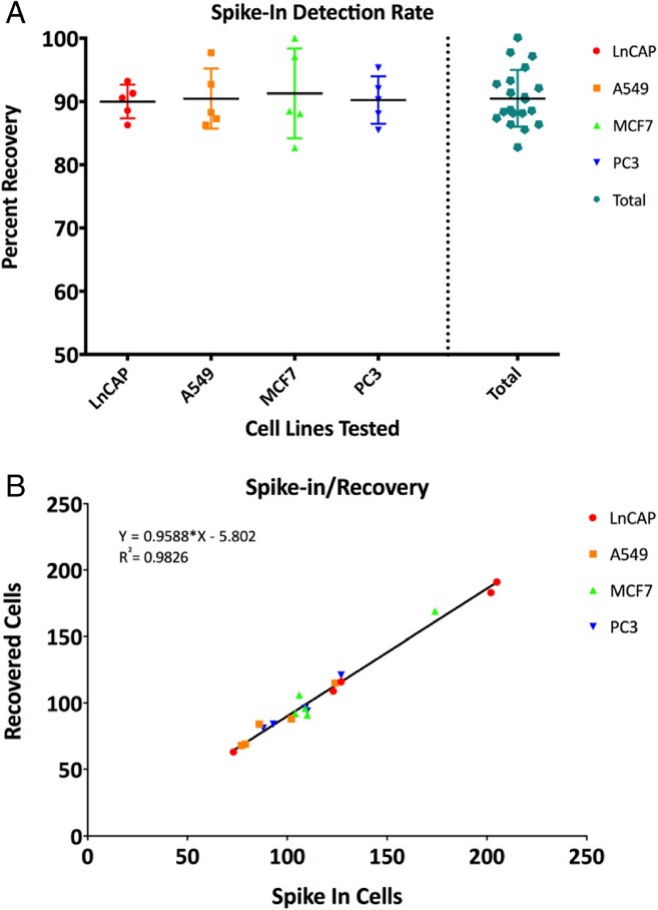
Recovery of known number of cells spiked into blood. **(A)** Scatter dot plot of spike-in cells with mean represented by the horizontal lines and standard deviation represented by vertical lines. **(B)** Linear regression analysis of recovered cells versus spiked in cells.

Tumor cell recovery counts were compared to the number of cells spiked into the blood in five replicates of each cell line. The mean recovery of mCTC detected by the AccuCyte – CyteFinder system ranged from 90% to 91% with an average recovery of 90.5% and standard deviation of 4.5 (Figure 4A). The mean percent recovery was 90.5 +/− 4.7 for A549, 90.0 +/− 2.6 for LNCaP, 90.2 +/− 3.7 for PC3, and 91.3 +/−7.1 for MCF7. The consistent recovery and narrow distribution indicates that the cell counts are highly reproducible over multiple samples and across cell lines of known high (LNCaP, MCF7) or low (PC3, A549) EpCAM expression. Linear regression analysis of the number of identified tumor cells against the number of spiked-in tumor cells produced a slope of 0.9588 and an intercept of 5.802 across all lines (Figure 4B). The correlation analysis of the results from all cell lines yielded an R^2^ value of 0.9826. There was no appreciable difference in recovery percentage across the range of cells spiked into the blood samples.

### Detection of single-digit numbers of spiked-in mCTC

Individually collected PC3 cells were spiked into 7.5 mL of whole blood using the CytePicker (see Methods) to determine the sensitivity of the AccuCyte – CyteFinder system to detect very low numbers of mCTC. Blood from 10 samples spiked with between 1 and 6 PC3 cells was processed to slides, stained and analyzed as described for the recovery experiments. The reviewer was blinded to the number of cells spiked-in. In 6 samples all cells were identified. In 3 samples, N – 1 cells were identified, and in 1 sample, N – 2 cells were identified, where N is the number of cells spiked in (Table 2). In total, 22 out of 27 (81%) mCTCs were identified, and in two of three experiments in which a single cell was spiked in, that one cell was identified. In one sample that had 3 mCTCs spiked in, 5 cells were identified. Upon review of the cells by a board-certified anatomic pathologist, two of the cells were determined to lack morphologic features of the mCTCs but did have features of squamous cells, consistent with venipuncture contaminants from skin. These results indicate that the AccuCyte – CyteFinder system is highly sensitive in identifying mCTCs at very low cell numbers and is capable of detection of a single cell in 7.5 mL of blood and underscore the value of high-resolution imaging for CTC classification.

**Table 2:** **Recovery of single-digit spike-in mCTCs**

Experiment	A	B	C	D	E	F	G	H	I	J
Number spiked-in mCTCs	1	1	1	2	2	3	3	3	5	6
Number mCTCs identified	1	1	0	2	2	3*	3	2	4	4

*In experiment F two additional cytokeratin-positive cells were identified by initial evaluator; these were determined on expert review to have morphology inconsistent with characterization as CTCs but consistent with being squamous cell contaminants from venipucture.

### CTC detection and characterization in breast cancer

**Figure 5:**
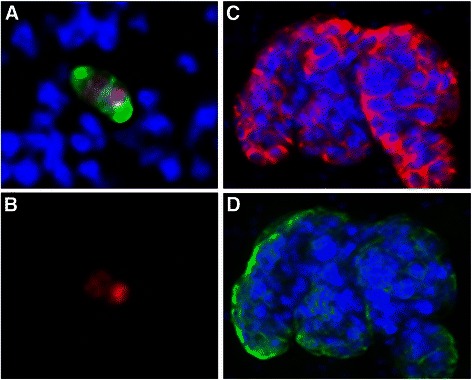
CTCs from a triple negative breast cancer patient. Blood was processed and scanned as described and after identification with the CyteMapper® software representative cells were re-imaged at 40X using the CyteFinder® Scanner. **(A)** CTC from a triple negative breast cancer patient stained with antibodies to cytokeratin (green), Ki67 (red) and DAPI (blue). Cell appears bi-nucleate, or may be near the end of cell division. **(B)** Same CTC shown in A, but with only Ki67 antibody staining in red. **(C, D)** Cluster of many CTCs with heterogeneous EpCAM (red) and cytokeratin (green) expression. DAPI was used to stain nuclei.

We applied the AccuCyte – CyteFinder system to the analysis of CTCs in breast cancer. Figure 5 shows cells from a patient with triple-negative breast cancer, having the characteristic cytoplasmic cytokeratin (5 A,D) and surface EpCAM (5C) staining and morphology of CTCs. We observed CTCs attached to one another in clusters (Figure 5C,D), which have been reported to be associated with worse outcome [16,17]. Examination of cell physiology markers in CTCs may be useful in investigation of therapeutic response. Cells that proliferate despite exposure to anti-cancer therapy by definition are not responding to the therapy, and thus may represent an important subset of cells for investigation. Since the AccuCyte – CyteFinder system is an open platform, we substituted an antibody against the proliferation antigen Ki-67 for the EpCAM antibody. A characteristic nuclear Ki-67 pattern was seen in cells identified as CTCs by cytokeratin staining (Figure 5A and B).

### AccuCyte-CyteFinder comparison to CellSearch

**Figure 6:**
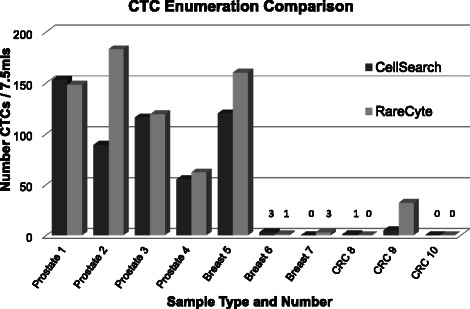
Comparison of CTC counts between AccuCyte – CyteFinder and CellSearch. 10 paired blood samples from patients with advanced prostate, breast or colorectal (CRC) cancer were processed independently using AccuCyte – CyteFinder or CellSearch systems to identify CTCs. In 3 samples counts equivalent (samples 1,3 and 4), in 3 samples AccuCyte – CyteFinder identified appreciably more CTCs than CellSearch (samples 2, 5 and 9), and in the remaining 4 samples numbers were very low by each method (3 CTCs or less).

Paired blood samples from a feasibility cohort of 10 patients with advanced breast, prostate or colorectal cancer were evaluated by both AccuCyte-CyteFinder and CellSearch CTC methods using similar criteria for identification of CTCs. Investigators at RareCyte were blinded to the CellSearch counts until after the results from both assays were documented. CTC counts fell into three categories: (1) equivalent, (2) very low by both methods, or (3) notably higher with AccuCyte – CyteFinder than CellSearch (Figure 6). These results are consistent with findings in model CTCs spiked into blood that yield equivalent numbers in cell lines that express high EpCAM levels, but higher AccuCyte – CyteFinder counts in lines that express low or absent EpCAM (data not shown). A rational explanation is that not all CTCs have sufficient EpCAM expression to be collected by immunomagnetic bead capture, but have adequate cytokeratin expression for identification of epithelial origin.

### Retrieval and molecular analysis of individual CTCs

**Figure 7:**
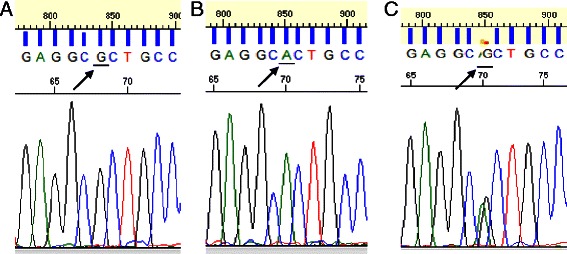
Single cell mutation detection after whole genome amplification. Sanger sequencing traces from SKBR3 cells show single nucleotide mutations in the TP53 gene. Shown above is the region of the gene containing the p.R175H mutation from PCR products derived from single cells spiked into blood, picked and amplified. CGC encodes the wild-type arginine and CAC encodes the mutant histidine found in SKBR3 cells. The nucleotides encoding these amino acids are underlined in the traces pictured above. **(A)** Wild type sequence from a WBC, **(B)** Mutant sequence from SKBR3 cells, **(C)** Mixture of mutant and wild type sequence most likely due to a contaminating WBC picked along with a SKBR3 cell.

To investigate whether genomic analyses can be performed on individual CTC that have been identified and retrieved using the methods described above, SKBR3 cells were spiked into a blood sample that was processed using the AccuCyte – CyteFinder system. mCTCs were identified and individually picked with the CytePicker (see Methods). These cells were then subjected to whole genome amplification (WGA). Using the WGA product, a region of the TP53 gene known to contain an R175H mutation in SKBR3 was amplified by PCR and the nucleotide sequence of this region was determined by Sanger sequencing. CGC encodes the arginine found in the wild type TP53 sequence and CAC encodes histidine, found in the mutant variant. Sequence from a picked WBC from the same blood sample the SKBR3 cells were spiked into revealed wild-type TP53 (Figure 7A). The mutation was clearly identified in SKBR3 (Figure 7B). Since SKBR3 cells are homozygous for this mutation, the sequence in Figure 7B verifies that a SKBR3 cell was picked from this slide independently. In Figure 7C the mutation was observed in the background of the wild type TP53 sequence; this likely indicates presence of an adjacent white blood cell in the WGA reaction.

### Whole exome sequencing and array CGH of SKBR3 mCTC

**Figure 8:**
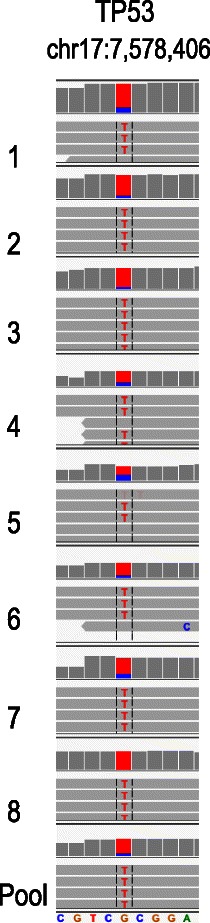
Whole exome sequencing of SKBR3 mCTCs. The chromosomal region containing the p.R175H mutation in TP53 is shown from whole genome amplification products from 8 individual cells and a pool of 5 cells that were picked from a slide processed as described. The nucleotides in red represent the mutation; wild type sequence is listed at the bottom. The mutation was identified in all samples.

A set of 9 SKBR3 WGA products from 8 individually picked mCTCs and a pool of 5 picked mCTCs from a spike-in blood sample were prepared for whole exome sequencing. Despite low read depth (15 – 30×) the TP53 R175H mutation was clearly demonstrated to be present in all 9 samples (Figure 8).

**Figure 9:**
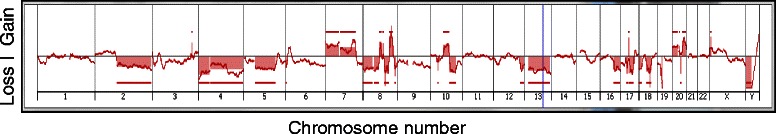
Array CGH of a single SKBR3 mCTC. Whole genome amplification product from a single picked SKBR3 mCTC was compared to a male control DNA sample by array-based hybridization. The horizontal axis shows chromosomal location. The vertical axis shows gains (above line) and losses (below line) for regions of each chromosome. Red lines above and below the graphed data designate regions of amplification or deletion.

In a separate experiment, the WGA product from a single SKBR3 mCTC picked from a spike-in blood sample was used for array-based comparative genomic hybridization (aCGH) using a reference male control DNA. The resulting karyotype was consistent with the published karyotype of this cell line (Figure 9). In addition to the expected sex chromosome mismatch, some of the expected findings that are consistent with these array results were: duplication of the majority of chromosome 7, 8q, proximal 10q, and chromosome 20; deletion of 8p, distal 10q, 16q, chromosome 18 (partial), and 19p. Findings that are not in the published karyotype were: deletion of 2q, chromosome 4, and the majority of 5q, which could have been acquired during culture.

## Discussion

Here we have presented a dual-technology platform for the identification and characterization of CTCs that is comprehensive, reproducible, and sensitive. Across 4 different cancer cell lines, more than 90% of mCTCs spiked into blood were consistently recovered in replicate experiments. Spike-in experiments using single-digit numbers of mCTCs demonstrated reproducible detection of the mCTCs with minimal loss and an experimental limit of detection of a single cell in 7.5 mL of blood. CTC counts in advanced stage cancer patients either exceeded or were similar to CellSearch CTC counts. Finally, individually visualized mCTCs were isolated using a retrieval device for the performance of molecular genetic analyses – including Sanger sequencing, whole exome sequencing, and array-based comparative genomic hybridization – that employed preparative single-cell whole genome amplification.

Our mCTC recovery experiments suggest that the fraction of the buffy coat that is collected using the AccuCyte system approaches 100%, since there is likely some decrease in CTC yield due to the staining and image analysis steps. In contrast, CPT tube and Ficoll-Hypaque® density-based separation methods are reported to have a maximum white blood cell yield of 60 – 80% and can be highly variable [18,19]. Thus, this platform ensures that virtually all of the CTCs present within a blood sample are deposited onto slides for microscopic analysis, regardless of their size or the expression of specific surface molecules.

In our experience, cytokeratin is a more reliable epithelial marker than EpCAM, which has variable expression across cell lines and even within clinical CTCs in a single cluster. Low EpCAM expression or EpCAM downregulation in CTCs have been cited as reasons for the inefficiency of EpCAM capture methods in certain cancer types [20-22]. In our study, the PC3 cell line expressing very low levels of EpCAM was recovered from spike in experiments as efficiently as cell lines expressing higher EpCAM levels. CyteFinder incorporates high-resolution (40× objective) cell imaging as an important tool for definitively classifying CTCs. For the low number spike-in experiments this tool was used to exclude cytokeratin-positive cells that lacked mCTC morphology.

At the center of the platform workflow is a staining step employing an automated IHC instrument (the Ventana Discovery® Ultra). Automated IHC instruments are common in histopathology laboratories worldwide, and their use simplifies workflow and reduces hands-on time requirements for sample processing. Moreover, they allow the application of various antibody reagents, providing for an “open” platform for CTC evaluation. These reagents may be to identify drug targets, such as Her2 for breast cancer, or non-epithelial phenotypic markers, such as CD146 and NG2 for melanoma, (data not shown) or physiologically meaningful biomarkers, such as Ki67, as demonstrated above. Markers for mesenchymal transformation or cancer differentiation are equally possible to incorporate. Recently we have demonstrated that CTC identification by the AccuCyte – CyteFinder system is independent of automated staining instrument; spike-in recovery of PC3 cells on the Dako Autostainer® Link 48 averaged 93%, and single digit spike-in limit of detection was also one cell in 7.5 mL (data not shown).

In a clinical feasibility cohort of advanced breast, prostate and colorectal cancer patients, AccuCyte – CyteFinder enumeration of CTCs compared favorably to the only FDA-cleared system for counting CTCs (CellSearch). This is consistent with the understanding that not all CTCs express sufficient EpCAM to be collected by immunomagnetic bead capture. Clinical application of the AccuCyte – CyteFinder system was also demonstrated in an evaluation of CTCs in a patient with advanced triple-negative breast cancer; here we demonstrated application of biomarkers for proliferation (Ki-67) and drug targeting (Her2), and observed cell clusters, which have been reported to be indicative of aggressive disease [16,17].

False positive CTC identification by AccuCyte – CyteFinder appears to be very low. In the spike-in recovery study of 20 samples, recovery rate was never greater than 100% (unlike some other CTC platforms), and in the single-digit spike-in experiments, only one sample had a higher CTC count than spike-in number, and these cells could be morphologically distinguished as non-malignant. Furthermore, in the ongoing comparison with CellSearch, we have evaluated numerous samples in which no cells have been found (data not shown). This is circumstantial evidence that false-positive identification is likely to be extremely rare. Formal studies of false-positive rate are important and will be performed in the future.

CTCs are increasingly regarded as windows through which to observe dynamic changes in the molecular biology of solid tumors. Retrieval of CTCs for molecular analysis will thus likely be an important aspect of future CTC technologies. We have demonstrated the use of an integrated single cell retrieval device, the CytePicker, that can routinely collect individual cells that are adherent to microscopic slides after identification with CyteFinder. The process is compatible with whole genome amplification of single cells, which then can be followed by various molecular genetic analysis methods. Here we have shown that both nested PCR followed by Sanger sequencing and whole exome sequencing identifed a known TP53 mutation in SKBR3 mCTCs, and that array-based comparative genomic hybridization confirmed the reported SKBR3 karyotype. Similar investigations are currently being undertaken in single CTCs from cancer patient samples.

## Conclusion

We have developed a comprehensive and sensitive dual-technology platform for flexible identification and characterization of CTCs on microscopic slides using established histopathology staining instruments. The platform has been successfully applied to longitudinal investigation of a patient with breast cancer on a clinical trial protocol and it can readily isolate single cells for sequencing and other genomic analyses. It thus permits the non-invasive and repeated accurate monitoring of therapeutic response and evolving cancer biology to enable personalized, molecularly-guided cancer treatment.

## Additional files

Additional file 1: Figure S1. Live cells were freshly prepared as a suspension and the nuclei were fluorescently pre-labeled before being drawn into a capillary tube (VitroTube®). The VitroTube was then scanned and cells were counted on a fluorescent microscope. Cells were expelled into blood sample by flushing with PBS and the VitroTube was rescanned and counted to obtain the net count of the cells added to the blood. (A) Fluorescent scan of Hoechst-stained cells in VitroTube (transmitted light overlay). (B) VitroTube on slide for scanning.
Additional file 2: Figure S2. Workflow for obtaining buffy coat using the AccuCyte® density-based separation system. A Add blood into AccuCyte Separation Tube containing float. B Centrifuge sample to separate constituent layers (top to bottom: plasma, buffy coat, red blood cells). C Apply sealing ring (arrow-head). D. Aspirate plasma (left); add high-density retrieval (HDR) fluid (right). E Insert EpiCollector™. F. Insert Transfer Tube pre-loaded with HDR fluid into EpiCollector. G. During second centrifugation, the HDR fluid displaces buffy coat cells which float to the top of the HDR fluid inside the Transfer Tube. H. Remove Transfer Tube containing buffy coat (Note: small amount of residual plasma remaining on float is collected, as well as small amount of red blood cells, due to placement of the sealing ring just below buffy coat – red blood cell interface).
Additional file 3: Figure S3. (A). Buffy coat spread onto slide prepared for automated staining. (B) Scan of slide after immunofluoresence staining comprised of 2419 individual 10x image panels. (C) The single panel (arrow in panel B) shows one such 10x image identified by the CyteMapper® software as containing a candidate CTC in box. (D) Cytokeratin positive CTC. Stains: DAPI (blue), CD45 (orange) and cytokeratin (green).
Additional file 4: Figure S4. CyteMapper® review software display of objects of interest from whole-slide scans. Candidate CTCs are identified by the analysis software using criteria such as signal intensity, object size and cellular morphology. Images are presented for characterization and enumeration of CTCs. Each channel can be viewed independently or in any combination and objects can be shown in greater detail to resolve subcellular details. The top row represents a fluorescent object found by the software that was rejected by the reviewer since morphology and staining are not consistent with classification CTC. The bottom row represents a candidate cell classified as a mCTC that is positive in all channels except for the channel containing CD45.
Additional file 5: Figure S5. CytePicker® single cell retrieval device. Candidate CTCs that are identified after CyteFinder® imaging can be picked using a software module that positions the needle tip over the cell of interest.
Additional file 6: Figure S6. Visual confirmation of cell removal with the CytePicker®. (A) 10X objective magnification image from CyteFinder of a model CTC (PC3 cell) stained with anti-cytokeratin antibody (green) in a background of white blood cells (blue nuclei) immediately before picking using the CytePicker module. (B) Image of the same region of the slide immediately after picking the model CTC. [Note: these images were made using the most recent version of the CyteFinder with CytePicker module].
