# Supplementary figures and images for: High-recovery visual identification and single-cell retrieval of circulating tumor cells for genomic analysis using a dual-technology platform integrated with automated immunofluorescence staining
Source: BMC Cancer. 2015 May 6;15:360. doi: 10.1186/s12885-015-1383-x (PMC4430903; doi:10.1186/s12885-015-1383-x)

## Slide 1
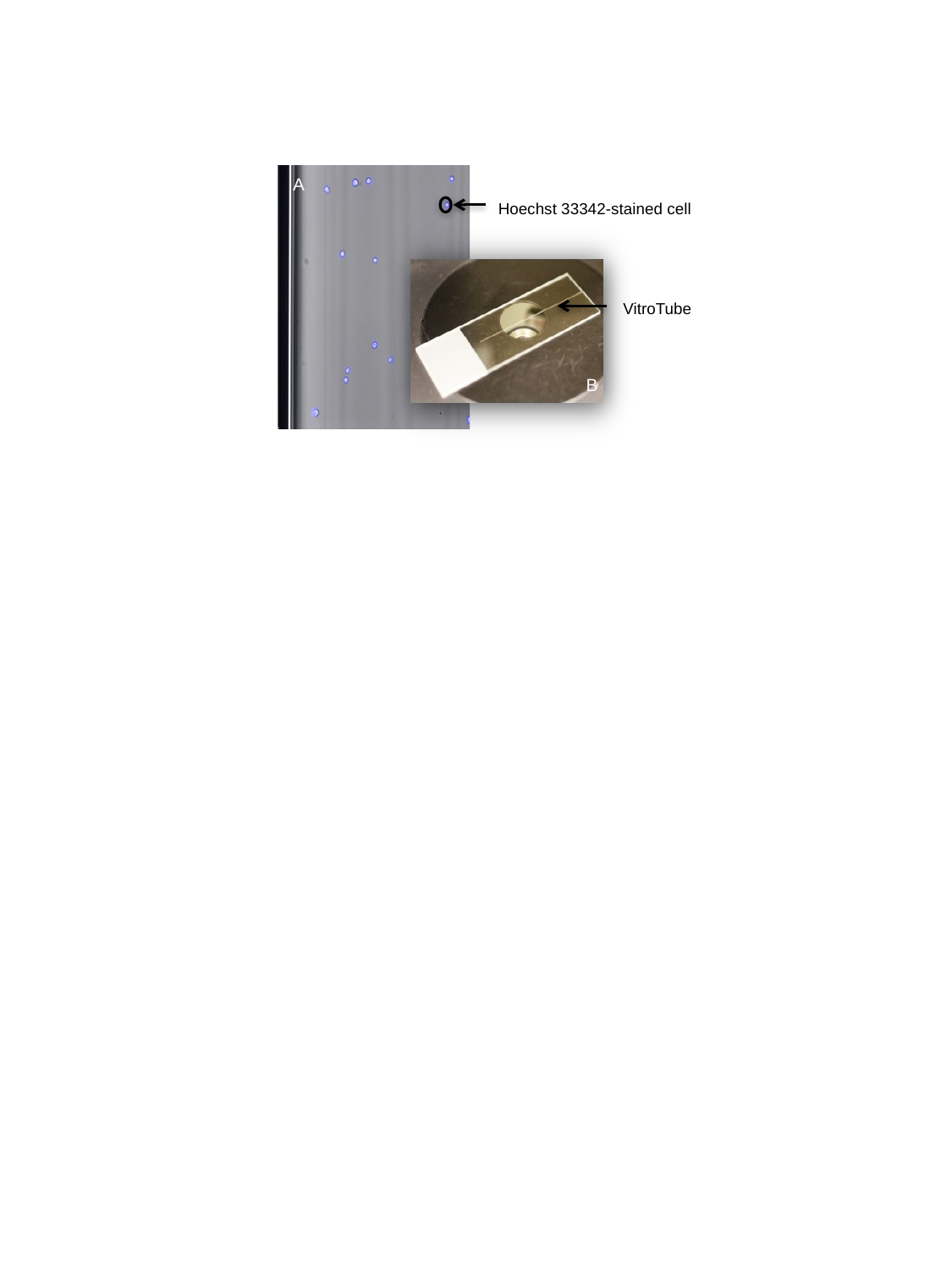

A
Hoechst 33342-stained cell
VitroTube
B

Supplement: Additional file 1: Figure S1. — Live cells were freshly prepared as a suspension and the nuclei were fluorescently pre-labeled before being drawn into a capillary tube (VitroTube®). The VitroTube was then scanned and cells were counted on a fluorescent microscope. Cells were expelled into blood sample by flushing with PBS and the VitroTube was rescanned and counted to obtain the net count of the cells added to the blood. (A) Fluorescent scan of Hoechst-stained cells in VitroTube (transmitted light overlay). (B) VitroTube on slide for scanning. [file 12885_2015_1383_MOESM1_ESM.pptx]

## Slide 1
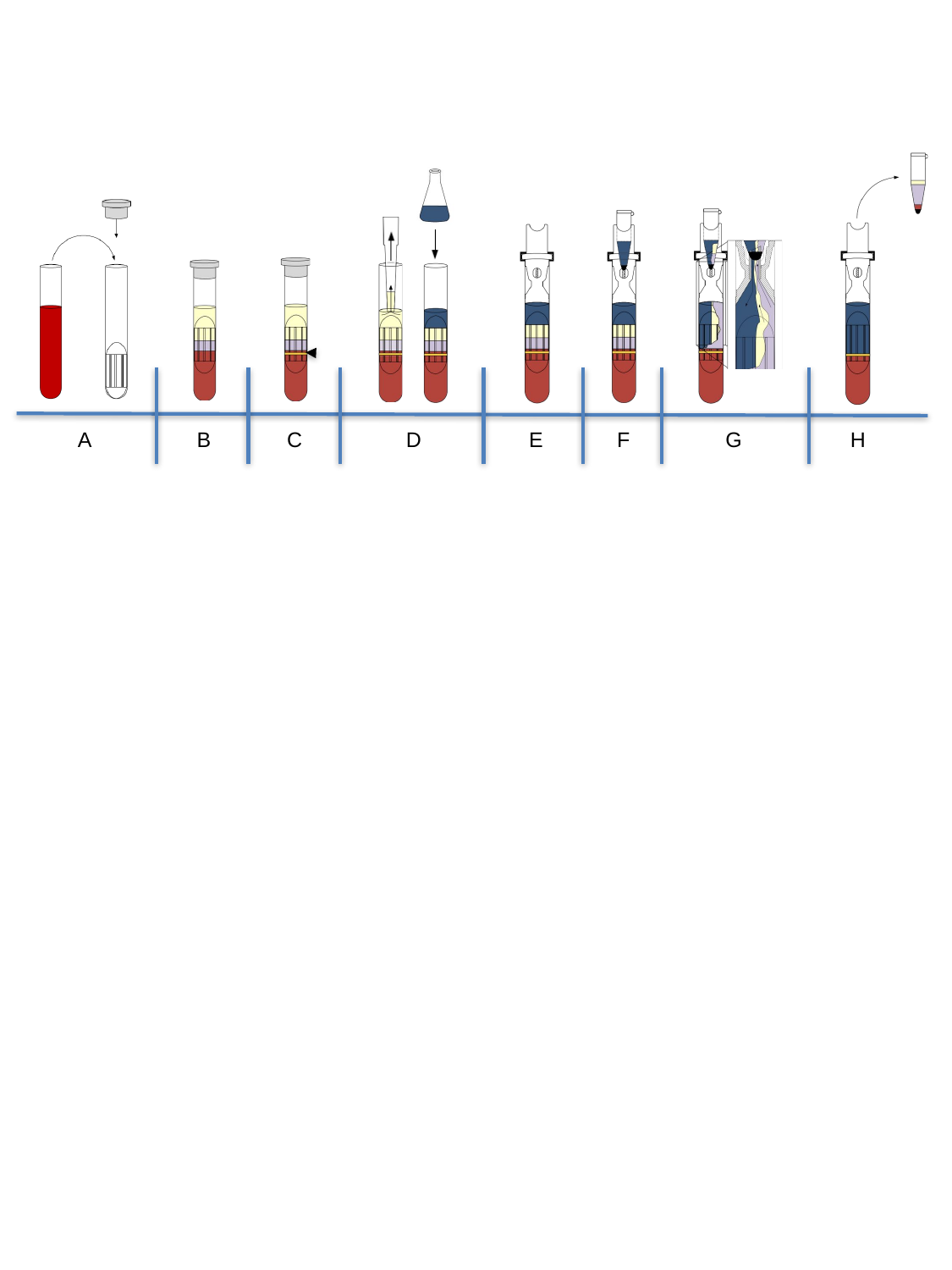

A
B
C
D
E
F
G
H

Supplement: Additional file 2: Figure S2. — Workflow for obtaining buffy coat using the AccuCyte® density-based separation system. A Add blood into AccuCyte Separation Tube containing float. B Centrifuge sample to separate constituent layers (top to bottom: plasma, buffy coat, red blood cells). C Apply sealing ring (arrow-head). D. Aspirate plasma (left); add high-density retrieval (HDR) fluid (right). E Insert EpiCollector™. F. Insert Transfer Tube pre-loaded with HDR fluid into EpiCollector. G. During second centrifugation, the HDR fluid displaces buffy coat cells which float to the top of the HDR fluid inside the Transfer Tube. H. Remove Transfer Tube containing buffy coat (Note: small amount of residual plasma remaining on float is collected, as well as small amount of red blood cells, due to placement of the sealing ring just below buffy coat – red blood cell interface). [file 12885_2015_1383_MOESM2_ESM.pptx]

## Slide 1
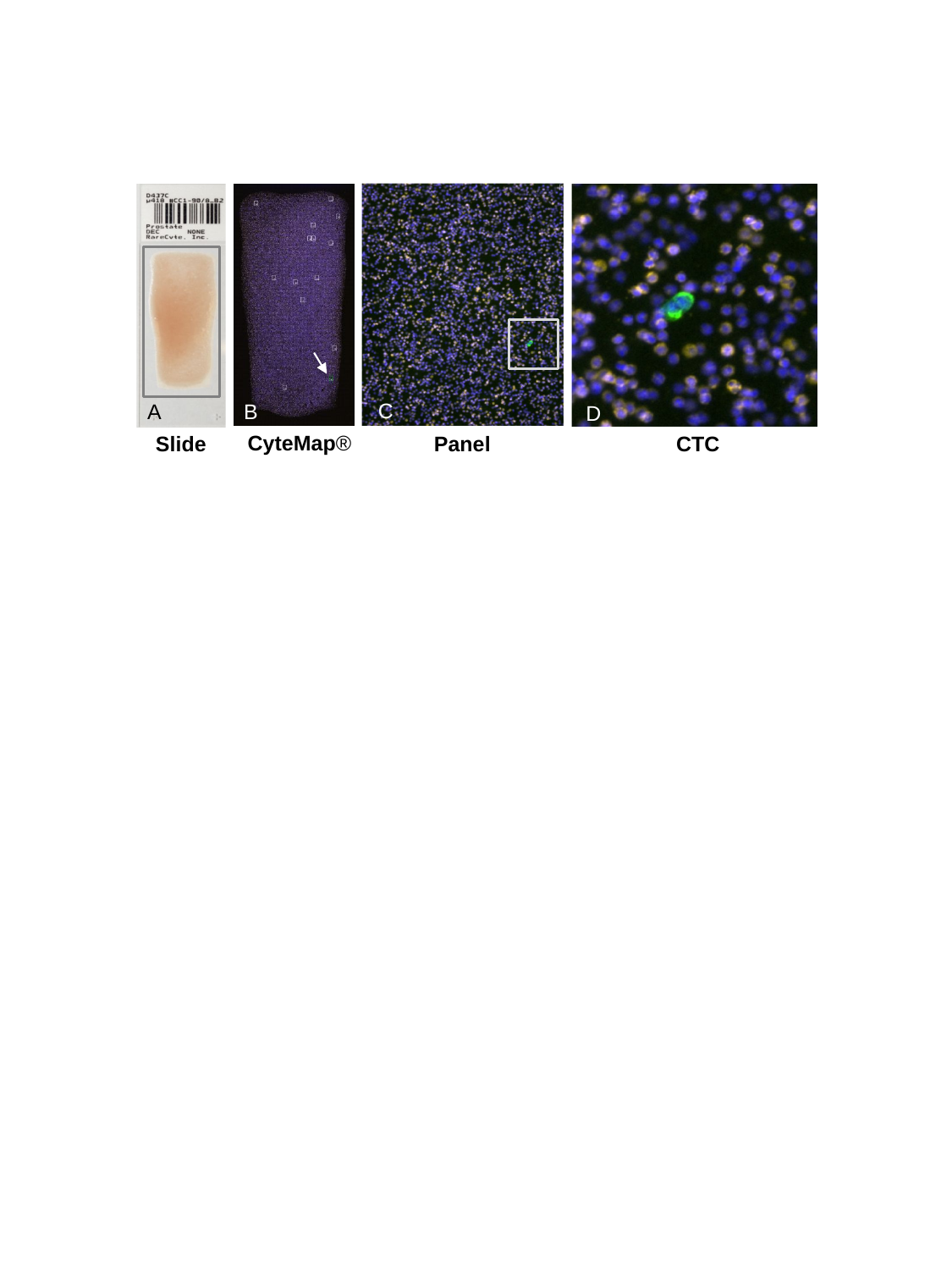

C
A
B
D
CyteMap®
Panel
Slide
CTC

Supplement: Additional file 3: Figure S3. — (A). Buffy coat spread onto slide prepared for automated staining. (B) Scan of slide after immunofluoresence staining comprised of 2419 individual 10x image panels. (C) The single panel (arrow in panel B) shows one such 10x image identified by the CyteMapper® software as containing a candidate CTC in box. (D) Cytokeratin positive CTC. Stains: DAPI (blue), CD45 (orange) and cytokeratin (green). [file 12885_2015_1383_MOESM3_ESM.pptx]

## Slide 1
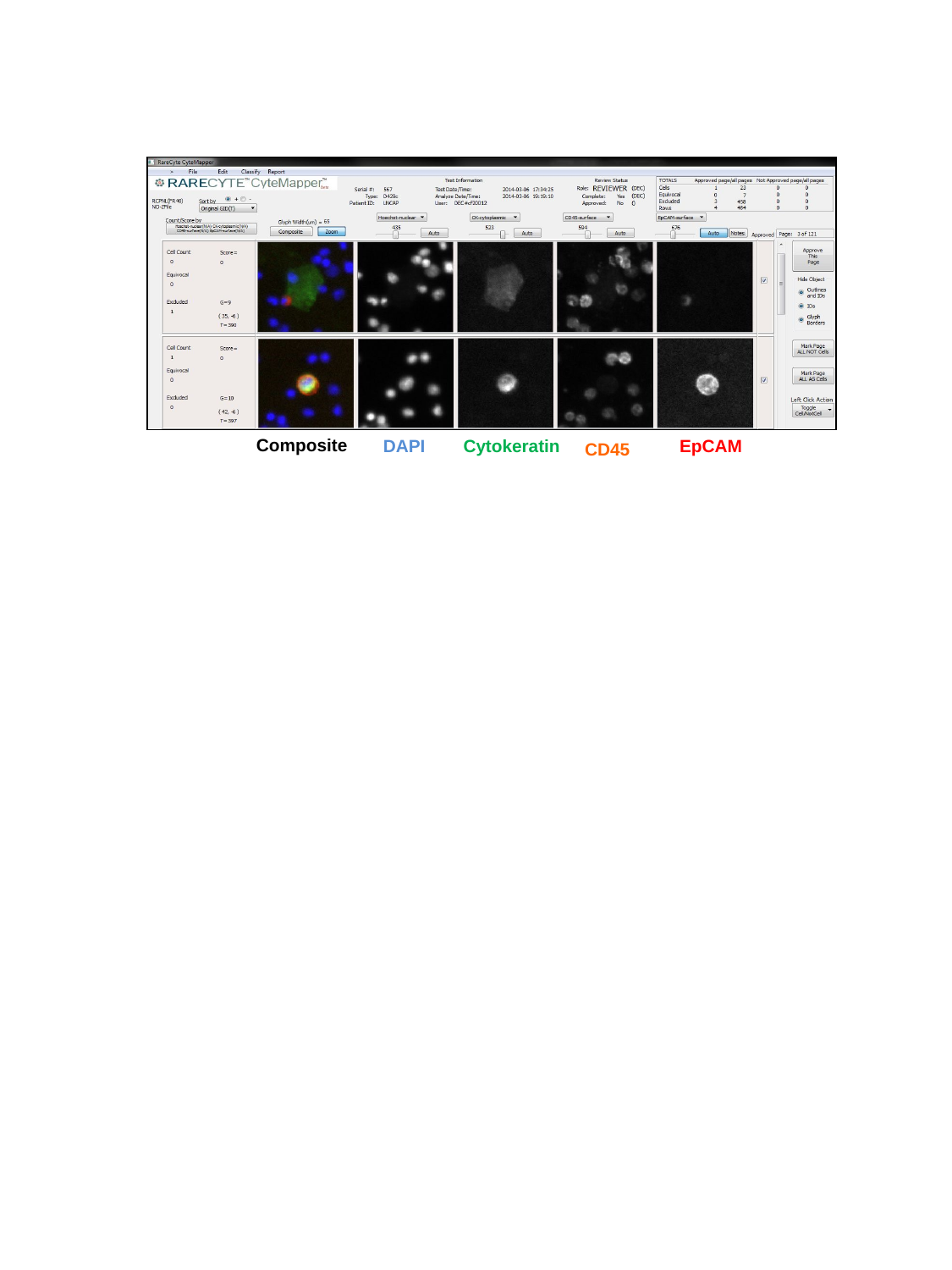

CD45
Composite
EpCAM
DAPI
Cytokeratin

Supplement: Additional file 4: Figure S4. — CyteMapper® review software display of objects of interest from whole-slide scans. Candidate CTCs are identified by the analysis software using criteria such as signal intensity, object size and cellular morphology. Images are presented for characterization and enumeration of CTCs. Each channel can be viewed independently or in any combination and objects can be shown in greater detail to resolve subcellular details. The top row represents a fluorescent object found by the software that was rejected by the reviewer since morphology and staining are not consistent with classification CTC. The bottom row represents a candidate cell classified as a mCTC that is positive in all channels except for the channel containing CD45. [file 12885_2015_1383_MOESM4_ESM.pptx]

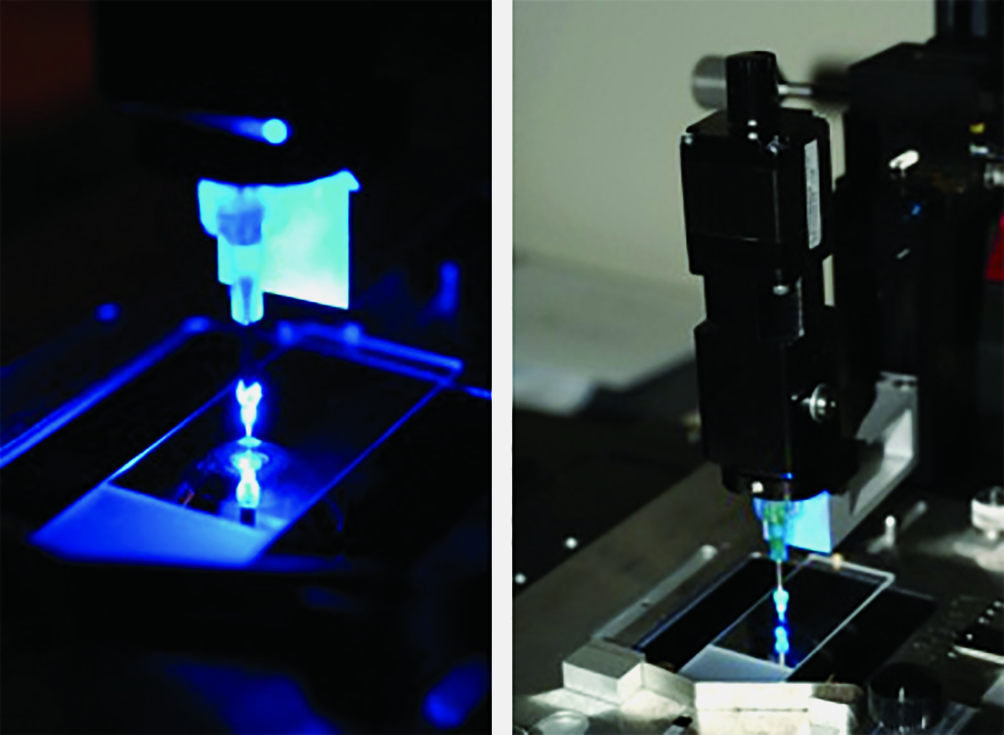

Supplement: Additional file 5: Figure S5. — CytePicker® single cell retrieval device. Candidate CTCs that are identified after CyteFinder® imaging can be picked using a software module that positions the needle tip over the cell of interest. [file 12885_2015_1383_MOESM5_ESM.tiff]

## Slide 1
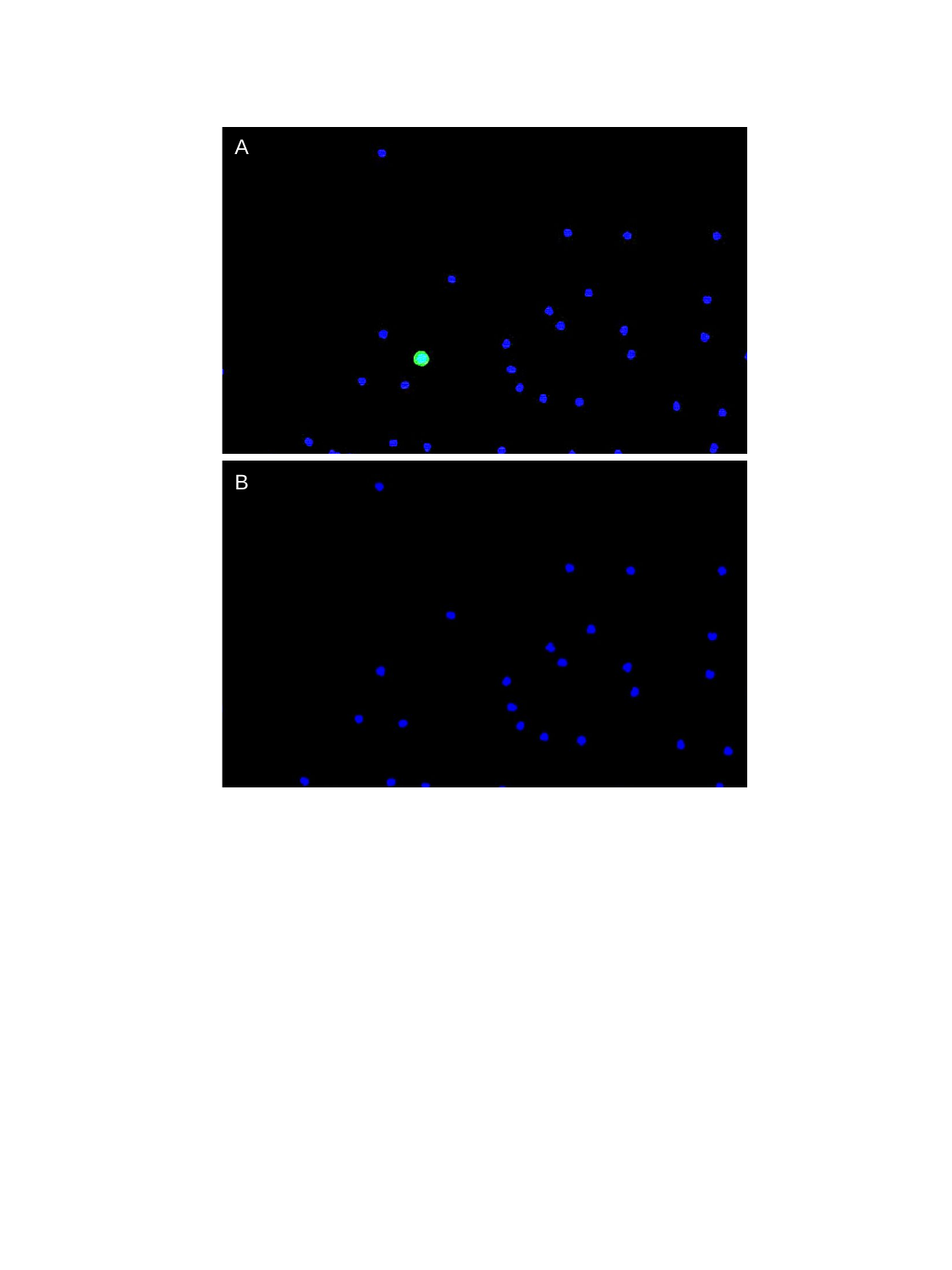

A
B

Supplement: Additional file 6: Figure S6. — Visual confirmation of cell removal with the CytePicker®. (A) 10X objective magnification image from CyteFinder of a model CTC (PC3 cell) stained with anti-cytokeratin antibody (green) in a background of white blood cells (blue nuclei) immediately before picking using the CytePicker module. (B) Image of the same region of the slide immediately after picking the model CTC. [Note: these images were made using the most recent version of the CyteFinder with CytePicker module]. [file 12885_2015_1383_MOESM6_ESM.pptx]
